# Effects of different sugar-lipid ratio diets on the occurrence of type 2 diabetes mellitus

**DOI:** 10.3389/fendo.2026.1768263

**Published:** 2026-04-23

**Authors:** Wenjie Sun, Linlin Jiang, Shanshan Tang, Xuedong An, Hangyu Ji, Fengmei Lian

**Affiliations:** 1 Guang’anmen Hospital, China Academy of Chinese Medical Sciences, Beijing, China; 2 Department of geriatrics, Shandong University of Traditional Chinese Medicine Affiliated Hospital, Jinan, Shandong, China; 3 Changchun University of Chinese Medicine, Changchun, Jilin, China

**Keywords:** dietary fat, dietary sugar, metabolomics, proteomics, type 2 diabetes mellitus

## Abstract

**Objective:**

Type 2 diabetes mellitus (T2DM) arises from sustained energy imbalance and macronutrient dysregulation. This study elucidates how distinct dietary sugar-to-lipid ratios modulate T2DM progression and delineates the underlying molecular mechanisms.

**Methods:**

Forty C57BL/6 mice were randomized into a control group (standard diet) and three high-energy cohorts with varying sugar-to-fat ratios (10% fat/70% carbohydrate; 45% fat/35% carbohydrate; 60% fat/20% carbohydrate). Body weight and fasting blood glucose were longitudinally monitored to assess obesity and T2DM onset. Following diagnosis, we analyzed serum metabolic profiles, insulin resistance, organ indices, and histopathology of the liver, pancreas, and white adipose tissue. Integrated proteomic and untargeted metabolomic analyses of liver tissue were employed to decode mechanistic pathways, with key targets validated via molecular assays.

**Results:**

Elevated dietary fat content dose-dependently accelerated obesity and T2DM onset, exacerbating glycolipid dysregulation, insulin resistance, hepatic steatosis, and adipose inflammation. Proteomic profiling revealed that differentially expressed proteins, primarily localized to the mitochondria, endoplasmic reticulum, and plasma membrane, were enriched in lipid, amino acid, and cofactor metabolism. Concurrently, metabolomics identified 4,276 hepatic metabolites with significant enrichment in glycerophospholipid and linoleic acid pathways. Integrated analysis demonstrated that high-fat diets disrupt systemic homeostasis by inducing coordinated perturbations in specific lipid metabolism networks. Validation confirmed that these diets suppressed mitochondrial markers (AMPK, PGC-1α, TFAM, NRF1) while dysregulating lipid regulators (upregulated PPAR-γ, downregulated PPAR-α).

**Conclusion:**

High-fat diets exert more severe metabolic detriment than other macronutrient configurations. This progression is driven by a dual interaction network involving mitochondrial dysfunction and lipid metabolic reprogramming, which collectively dismantle systemic metabolic homeostasis.

## 1 Introduction

Type 2 diabetes mellitus (T2DM) represents a critical non-communicable disease posing a severe threat to global health (1). While clinically defined by persistent hyperglycemia, T2DM is increasingly characterized as a complex metabolic syndrome driven by chronic energy imbalance and modern dietary shifts (2). Consequently, risk factors are predominantly linked to macronutrient maladaptation, specifically the consumption of high-energy diets rich in sugars and fats.

Research examining dietary fat indicates that excessive intake exacerbates T2DM risk factors, including dyslipidemia, obesity, and hypertension (3). A positive correlation exists between the proportion of energy derived from fat and key metabolic indices such as body mass index (BMI), total cholesterol (TC), and low-density lipoprotein cholesterol (LDL-C), which collectively heighten T2DM susceptibility (4). Longitudinal data from Chinese adults confirm that shifts toward high-fat diets are significantly associated with obesity, diabetes, and all-cause mortality (5). Furthermore, high fat intake is directly implicated in T2DM incidence (6–8). Conversely, the relationship between carbohydrate intake and T2DM is not linear. Although high-sugar diets are established risk factors (9, 10), carbohydrate energy contribution exhibits a U-shaped association with disease onset; a moderate intake range minimizes risk, whereas consumption falling below or exceeding this threshold accelerates T2DM development (11).

Indisputably, excessive consumption of fats or carbohydrates induces a positive energy balance, thereby driving metabolic pathologies such as obesity and T2DM. While both high-fat diets (HFD) and high-sugar diets (HSD) are implicated in the pathogenesis of T2DM, total caloric surplus is often posited as the predominant driver of metabolic abnormalities. However, a critical unresolved question is whether a high-fat regimen exerts a more deleterious effect on T2DM progression compared to a high-sugar profile. To date, a consensus remains elusive; existing evidence suggests that elevated ratios of either macronutrient can precipitate disease onset, yet their comparative impact warrants further clarification.

Mechanistically, the differential impacts of high-fat and high-sugar diets on T2DM pathogenesis are rooted in systemic metabolic partitioning and appetite regulation. Fundamentally, chronic overconsumption of sugars and lipids disrupts substrate oxidation preferences, leading to ectopic fat deposition, a core driver of insulin resistance (IR) (12). According to the physiological oxidation hierarchy, humans preferentially oxidize carbohydrates; thus, increased carbohydrate intake stimulates a corresponding rise in carbohydrate oxidation (4). In contrast, excessive dietary fat fails to promote its own oxidation in the short-to-medium term, leading to greater adipose accumulation compared to isocaloric carbohydrate intake (13). Chronically, this metabolic inflexibility favors lipid storage (14). Furthermore, high-fat intake compromises the regulation of the feeding center, resulting in chronic positive energy balance and obesity-related IR (15). Unlike carbohydrates, dietary fat is a nutrient consistently associated with hyperphagia due to its high energy density and low oxidative rate, which facilitate passive overconsumption (16). Additionally, fat exerts a weaker satiety effect than carbohydrates, further propagating excessive energy intake (17). Consequently, a high-fat dietary pattern is intrinsically more prone to inducing lipid storage and systemic metabolic dysregulation, thereby amplifying the risk of T2DM compared to high-carbohydrate regimens.

Mitochondria function as a central metabolic nexus, integrating glucose and lipid metabolism to modulate hepatic lipid accumulation. Multi-omics analyses reveal that impaired mitochondrial biogenesis triggers a coordinated proteomic response, fundamentally disrupting lipid and glycoprotein homeostasis (18). As a master regulator of mitochondrial biogenesis, Peroxisome proliferator-activated receptor γ coactivator 1alpha (PGC-1α) orchestrates lipid metabolism and IR (19, 20). PGC-1α exerts a dual regulatory function: it potentiates lipogenesis by activating the liver X receptor α (LXRα)-mediated Fatty Acid Synthase (FASN) promoter (21), while concurrently promoting mitochondrial β-oxidation through co-activation of PPAR-α and PPAR-δ (22). Underscoring this mechanism, sortilin in adipocytes has been shown to upregulate mitochondrial acyl-CoA synthetase long-chain family member 1 and activate the Adenosine 5’-monophosphate (AMP)-activated protein kinase (AMPK)/PGC-1α signaling pathway, thereby mitigating high-fat diet-induced obesity and IR (23). Clinically, T2DM manifests primarily as IR in early stages, progressing to pancreatic β-cell dysfunction (24). Thus, mitochondrial dysfunction-driven lipid metabolic dysregulation constitutes a critical pathway linking macronutrient excess to T2DM.

Existing epidemiological evidence strongly suggests that an increased proportion of dietary energy derived from fat is associated with an elevated risk of obesity and chronic conditions such as T2DM. While high-sugar and high-fat diets are both implicated in T2DM pathogenesis, clinical studies have yielded discordant findings regarding their comparative impact, largely due to the difficulty of isolating the specific sugar-to-fat ratio in human populations. Consequently, controlled animal models are essential to rigorously evaluate the effects of distinct macronutrient ratios on disease latency and incidence. Mechanistically, we hypothesize that a high-fat dietary pattern predisposes the liver to lipid storage more severely than high-sugar regimens. As the central metabolic engine processing both substrates, mitochondria are critical; their dysfunction is posited as the initiating factor in lipid metabolic imbalance. The resulting lipotoxicity may disrupt insulin signaling and exacerbate IR, thereby driving T2DM progression. To validate this hypothesis and clarify the underlying molecular pathways, this study employs an integrated multi-omics approach.

## 2 Methods

### 2.1 Animals and diet

Forty 7-week-old male C57BL/6J mice (20 ± 2 g) were obtained from Beijing Huafukang Bioscience Co., Ltd. Animals were maintained in the specific pathogen-free (SPF) facility of the Institute of Basic Theory, China Academy of Chinese Medical Sciences, under controlled environmental conditions (18–22 °C, 40–70% relative humidity, 12-h light/dark cycle). Mice were housed five per cage with ad libitum access to water and diet. All experimental protocols complied with ethical standards and were approved by the Animal Care and Use Committee of Guang’anmen Hospital (Ethics Approval No.: IACUC-GAMH-2023-025-01).

During the acclimatization period, mice were fed a standard maintenance diet (Product No.: 1016706714625204224) sourced from Beijing Ke’ao Xieli Feed Co., Ltd. Subsequently, experimental groups received specific diets from Research Diets (USA) with varying macronutrient compositions: a 60% fat/20% carbohydrate diet (D12492), a 45% fat/35% carbohydrate diet (D12491), and a 10% fat/70% carbohydrate diet (D12450B). Detailed dietary compositions and energy ratios are provided in **Supplementary Table 1**.

### 2.2 Experimental design

Mice were randomized into four groups (n = 10) using a random number table: a control (CON) group fed a normal diet, and three experimental cohorts (M10, M45, M60) receiving diets with 10%, 45%, and 60% fat content, respectively. Body weight, body length, and fasting plasma glucose (FPG) were monitored bi-weekly. The intervention concluded at 12 weeks, coinciding with the attainment of T2DM diagnostic criteria in the experimental groups. At the endpoint, overnight-fasted mice were anesthetized with intraperitoneal pentobarbital sodium (40 mg/kg; 0.4% solution) and sacrificed via cardiac exsanguination. The liver, pancreas, and epididymal white adipose tissue (eWAT) were harvested and weighed to calculate organ indices. Tissue samples were aliquoted: one portion was fixed in 4% paraformaldehyde or electron microscopy fixative for histological analysis, while the remainder was snap-frozen on dry ice and stored at -80 °C for omics and molecular assays.

### 2.3 Assessment of obesity and T2DM

#### 2.3.1 Lee’s index

Following the 12-week intervention, body length (nose to anus) was measured. Lee’s index, an indicator of obesity (25), was calculated as: body weight (g)^(1/3)/body length (cm).

#### 2.3.2 Obesity incidence

Body weight was measured biweekly. Obesity was defined as an individual’s weight exceeding the normal control group average by >20%. Incidence was calculated as (obese mice/total group mice) × 100%.

#### 2.3.3 T2DM incidence

Fasting blood glucose (FBG) was measured biweekly. T2DM Incidence was defined as FBG > 11.1 mmol/L on two consecutive measurements. Incidence was calculated as (diabetic mice/total group mice) × 100%.

### 2.4 Oral glucose tolerance test

After 12 weeks of dietary intervention, mice underwent an oral glucose tolerance test (OGTT). Following a 6-h light-phase fast, baseline glucose was recorded, and a glucose load (2 g/kg) was administered via oral gavage. Blood glucose concentrations were monitored from tail vein samples at 30, 60, 90, and 120 min post-administration using an ACCU-CHEK glucometer (Roche, Basel, Switzerland; previously validated linear range: 4–25 mmol/L) (24). The area under the curve (AUC) for glucose excursion was calculated according to the following formula:


$$\text{AUC }=\;0.5\;\times \;\left(\text{B}{\text{G}}_{0}\;+\text{ B}{\text{G}}_{30}\right)/2\;+\;0.5\;\times \;\left(\text{B}{\text{G}}_{30}\;+\text{ B}{\text{G}}_{60}\right)/2\;+\;1\;\times \;\left(\text{B}{\text{G}}_{60}\;+\text{ B}{\text{G}}_{120}\right)/2,$$

where BG represents blood glucose (mmol/L) at the indicated time points.

### 2.5 Biochemical examinations

Serum samples from each group of mice were analyzed using a fully automated biochemical analyzer (Toshiba Fully Automated Biochemical Analyzer, Japan; Beckman AU5800 Automated Chemiluminescence Analyzer). The specific biochemical parameters measured included cholesterol (CHO), triglycerides (TGs), high-density lipoprotein cholesterol (HDL-C), and LDL-C, glycated serum protein (GSP).

The triglyceride glucose index (TyG) was calculated using the following formula: TyG = ln[fasting TG (mg/dL) × FBG (mg/dL)/2].

### 2.6 Enzyme-linked immunosorbent assay

Serum levels of fasting insulin (FINS), inflammatory cytokines, and adipokines were measured using ELISA. FINS was quantified with a commercial insulin assay kit (EMINS; Thermo Fisher Scientific, USA). The concentrations of tumor necrosis factor-α (TNF-α; EMC102a), interleukin-1β (IL-1β; EMC001b), and interleukin-6 (IL-6; E-EL-M004) were determined using corresponding ELISA kits (NeoBioscience, China for TNF-α and IL-1β; Elabscience, China for IL-6). Additionally, serum levels of adiponectin (ED295; Lianke Bio, China) and leptin (EK-297; Lianke Bio, China) were analyzed using their respective ELISA kits. All procedures were performed in strict accordance with the manufacturer’s instructions to ensure consistency and accuracy.

Additionally, the homeostasis model assessment of insulin resistance (HOMA-IR) and β-cell function (HOMA-β) were calculated as follows: HOMA-IR = [FBG (mmol/L) × FINS (μU/mL)]/22.5, and HOMA-β = [20 × FINS (μU/mL)]/[FBG (mmol/L) − 3.5].

### 2.7 Histopathological morphology

#### 2.7.1 Hematoxylin and eosin staining

Liver, pancreas, and adipose tissue specimens were fixed in 4% neutral-buffered formalin for 24 h, dehydrated, cleared, and embedded in paraffin. Serial sections (3–5 μm thick) were deparaffinized, rehydrated, and subjected to hematoxylin and eosin (H&E) staining. The protocol involved hematoxylin staining (3–5 min), brief differentiation in 1% acid alcohol, bluing, and counterstaining with 0.5–1% eosin (1–3 min). Following stepwise dehydration and clearing, slides were mounted with neutral medium. Histological morphology was evaluated via microscopic examination, with all procedures performed in triplicate.

#### 2.7.2 Oil Red O staining

Frozen sections of liver and adipose tissue were air-dried and fixed for 15 min. Oil Red O working solution (saturated stock: distilled water, 6:4; incubated overnight at 4 °C) was filtered prior to use. Sections were stained in the dark (8–10 min), differentiated sequentially in 60% isopropanol (3 s and 5 s), and washed. Nuclear counterstaining was performed with hematoxylin (3–5 min), followed by differentiation and bluing. Slides were mounted in glycerin gelatin and visualized microscopically, with all procedures replicated in triplicate.

### 2.8 Proteomics analysis of liver tissue

Proteomic analysis was conducted by Shanghai Majorbio Bio-Pharm Technology Co., Ltd. Total liver protein was extracted using lysis buffer (8 M urea, 1% SDS, protease inhibitors) and quantified via BCA assay. For digestion, proteins were reduced with TCEP, alkylated with iodoacetamide, and digested overnight with trypsin. Following desalting and quantification, peptides were analyzed via data-independent acquisition (DIA) on a Vanquish Neo UPLC system coupled to an Orbitrap Astral mass spectrometer (Thermo Scientific). Separation was performed on a µPAC column using an 8-min gradient, with data acquired in positive ion mode (m/z 100–1700).

Raw DIA data were processed using Spectronaut™ (version 19) against the UniProt Mus musculus database. Differentially expressed proteins (DEPs) were identified based on a fold change (FC) > 2 and *P* < 0.05 (Student’s t-test). Functional analysis, including Gene Ontology (GO) annotation and Kyoto Encyclopedia of Genes and Genomes (KEGG) pathway enrichment, was performed using the Majorbio Cloud Platform (https://cloud.majorbio.com/).

### 2.9 Metabolomics analysis of liver tissue

Metabolomic analysis was conducted by Shanghai Majorbio Bio-Pharm Technology Co., Ltd. Liver tissues (50 mg) were extracted with 400 μL of cold solvent (acetonitrile:methanol:water, 2:2:1, v/v/v) containing internal standards. Following homogenization, ultrasonication (ice-water bath), and centrifugation (13,000×g, 15 min, 4 °C), supernatants were evaporated under nitrogen and reconstituted in 120 μL acetonitrile:water (1:1, v/v). Quality control (QC) samples were prepared by pooling equal aliquots of all samples. LC-MS/MS analysis was performed on a Vanquish UPLC system (Thermo Fisher Scientific) coupled to a TripleTOF 6600 (SCIEX). Separation utilized an HSS T3 column (100 mm×2.1 mm, 1.8μm) at 40 °C with a flow rate of 0.40 mL/min. The mobile phase comprised 0.1% formic acid in water (A) and 0.1% formic acid in acetonitrile:isopropanol (1:1) (B), delivered over a 16-min gradient. MS data were acquired in positive and negative ion modes (scan range m/z 50–1000).

Raw data were processed using Progenesis QI for baseline correction, alignment, and integration. Metabolites were identified via HMDB and METLIN databases. Multivariate analysis (OPLS-DA) was employed to screen differential metabolites based on Variable Importance in Projection (VIP) ≥ 1.0, FC > 1, and *P* < 0.05 (Student’s t-test). Pathway enrichment was assessed using the KEGG database.

### 2.10 Immunohistochemical staining

Liver sections were deparaffinized, rehydrated, and subjected to antigen retrieval. Endogenous peroxidase activity was quenched with 3% H_2_O_2_. Following serum blocking, sections were incubated overnight at 4 °C with primary antibodies against NRF1 (83092-1-RR), PPARγ (66936-1-Ig), PGC-1α (66369-1-Ig), PPARα (66826-1-Ig), and TFAM (22586-1-AP)—all sourced from Proteintech—and β-actin (TDY041; TDY, China). Subsequently, sections were incubated with HRP-conjugated secondary antibodies (50 min, room temperature). Immunoreactivity was visualized using diaminobenzidine (DAB), with reaction termination upon microscopic confirmation. Slides were counterstained with hematoxylin, dehydrated, cleared, and mounted for microscopic analysis.

### 2.11 Quantitative real-time PCR

Total RNA was extracted from liver tissues using RNAisoPlus reagent (15596026CN; Invitrogen, USA) and reverse-transcribed into cDNA using a HiFiScript cDNA Synthesis Kit (CW2569; CWBIO, China). Quantitative PCR was performed using UltraSYBR Mixture (CW2602; CWBIO, China) on a StepOne Plus system (Applied Biosystems, USA). The relative mRNA expression levels were normalized to β-actin and calculated using the 2−ΔΔCT method. Primer sequences are listed in **Supplementary Table 2**.

### 2.12 Statistical analysis

Statistical analyses were performed using GraphPad Prism (version 10.4.0), with data expressed as mean ± standard deviation (SD). Normality was verified via the Shapiro–Wilk test. Group comparisons were conducted using unpaired Student’s t-tests for two groups and one-way analysis of variance (ANOVA) followed by Tukey’s *post-hoc* test for multiple groups. The cumulative incidence of obesity and T2DM was evaluated using Kaplan–Meier survival analysis and the log-rank test. A P-value < 0.05 was considered statistically significant. All experiments were independently replicated at least three times.

## 3 Results

### 3.1 A higher fat diet accelerates the onset risk of obesity and T2DM events in mice

**Figure 1:**
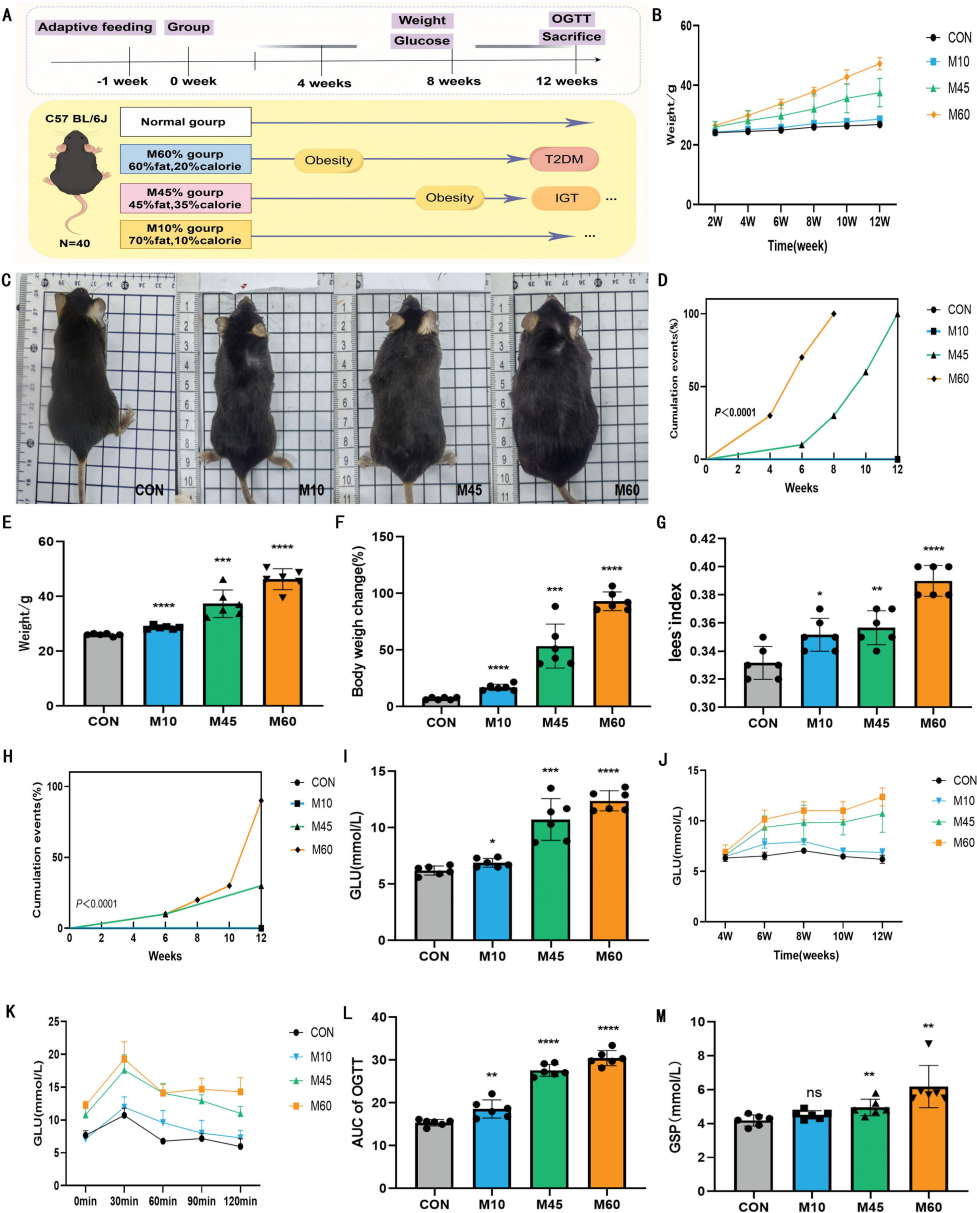
Obesity and T2DM were observed in mice fed with different carbohydrate-fat ratio diets. **(A)** Experimental workflow. **(B)** Weight change trends of mice at different time points after feeding different diets. **(C)** Representative appearance images of mice in each group. **(D)** Kaplan-Meier plots of the occurrence of obesity events in each group of mice (*n* = 10). **(E)** Body weight of mice in each group after 12 weeks of intervention (*n* = 8). **(F)** Body weight change rate of mice in each group (*n* = 8). **(G)** Lee’s index of mice in each group after 12 weeks of intervention (*n* = 8). **(H)** Kaplan-Meier plots of T2DM events in each group of mice (*n* = 10). **(I)** Fasting blood glucose levels change trend of mice in each group (*n* = 8). **(J)** Fasting blood glucose levels in different diets groups after 12 weeks of intervention (*n* = 8). **(K, L)** Oral glucose tolerance test curve and area under the curve (AUC) for each group (*n* = 6). **(M)** The levels of glycosylated serum protein in the mice of each group at the 12-week intervention period (n = 6).In statistical analysis, all values are expressed as mean ± SD. * indicates statistical difference between the model and control groups, ^*^P < 0.05, ^**^ *P* < 0.01, ^***^P < 0.001.

Contrary to the CON group, which maintained baseline trajectories, mice fed experimental diets demonstrated significant weight gain. At the 12-week endpoint, morphometric parameters—including body weight, weight gain rate, and Lee’s index—were significantly elevated in the M60 group compared to the M45 and M10 groups, indicating a direct proportionality to dietary fat content. Time-course analysis of obesity events identified a distinct hierarchy in onset latency: significant weight divergence from CON occurred at Week 2 for M60 (*P* < 0.01), Week 6 for M45, and Week 8 for M10. Although the M10 group exhibited greater weight gain than CON, it sustained a 0% obesity incidence, suggesting a prolonged latency period compared to the rapid obesity induction in the M60 group (**Figures 1B–G**).

Regarding glucose homeostasis, the high-fat diets induced significant hyperglycemia relative to the stable normoglycemia of the CON group. Elevated FBG emerged in all model groups by week 6. By the 12-week endpoint, both FBG and GSP levels were highest in the M60 group, correlating positively with dietary fat intake. OGTT confirmed this trend, with AUC values significantly increased in a fat-content-dependent manner (M60 > M45 > M10 > CON), reflecting varying degrees of glucose intolerance. Survival analysis further demonstrated that the M60 diet precipitated T2DM onset earlier and resulted in a significantly higher cumulative incidence compared to the moderate- and low-fat groups (**Figures 1H–M**).

### 3.2 A higher fat diet exacerbates insulin resistance and metabolic dysregulation in mice

**Figure 2:**
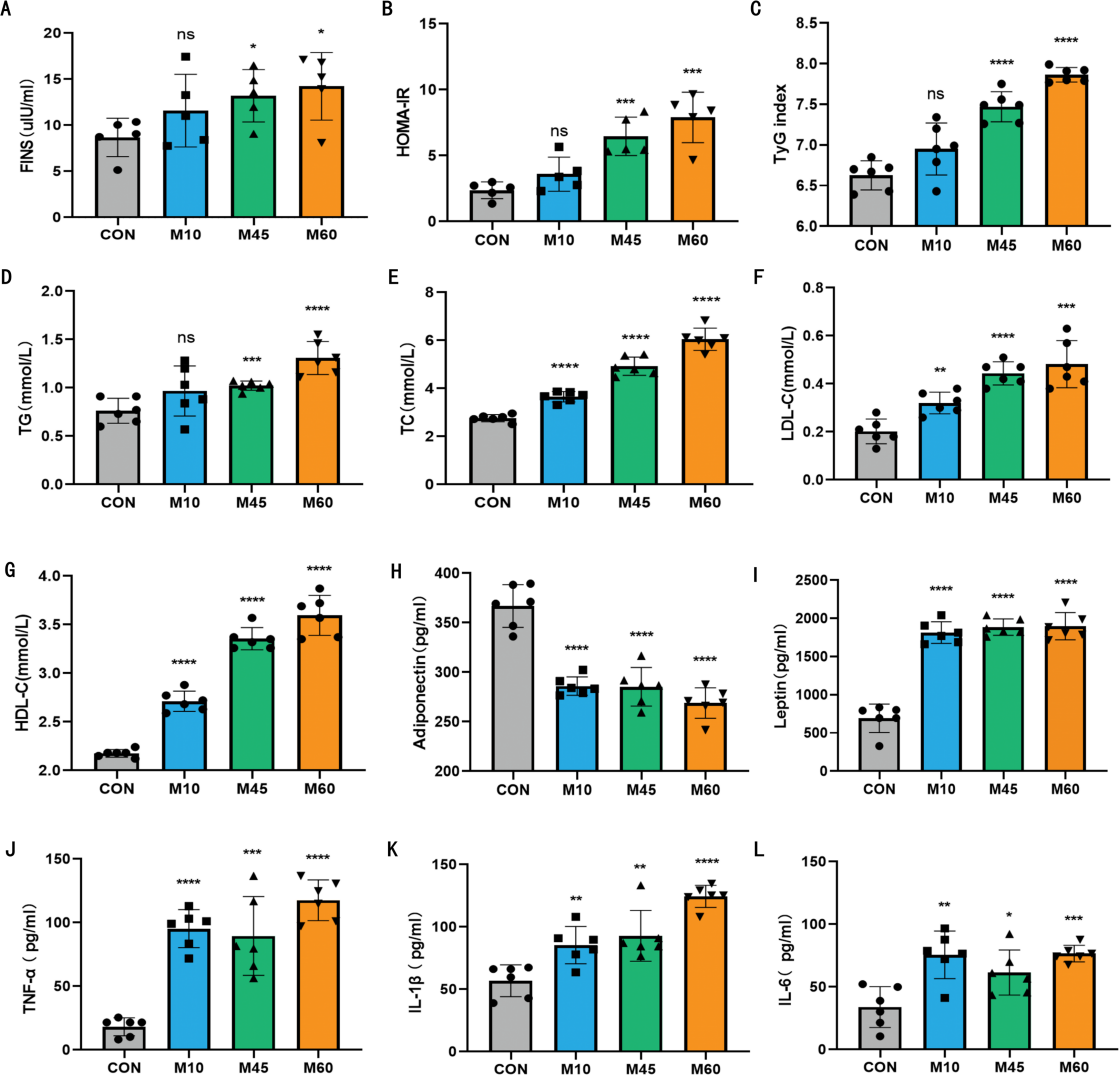
Insulin resistance and metabolic profile of mice treated with different carbohydrate-fat ratio diets. **(A)** Fasting serum insulin levels(FINS) in mice (n = 6). **(B)** Homeostasis Model Assessment-Insulin resistance (HOMA-IR) in mice (n = 6). **(C)** Triglyceride glucose (TyG) index in mice (n = 6). **(D–G)** Serum triglyceride (TG), total cholesterol (TC), low-density lipoprotein cholesterol (LDL-C), and high-density cholesterol (HDL-C) levels (n = 6). **(H)** Serum adiponectin levels in mice (n = 6). **(I)** Serum leptin levels in mice (n = 6). **(J)** Serum TNF-α levels in mice (n = 6). **(K)** Serum IL-1β levels in mice (n = 6). **(L)** Serum IL-6 levels in mice (n = 6). In statistical analysis, all values are expressed as mean ± SD. In statistical analysis, all values are expressed as mean ± SD. * indicates statistical difference between the model and control groups, ^*^P < 0.05, ^**^ *P* < 0.01, ^***^P < 0.001.

By week 12, the M60 and M45 groups exhibited significantly elevated FINS and HOMA-IR levels compared to controls (*P* < 0.05), following the severity gradient M60 > M45. Although the M10 group showed a non-significant upward trend in these markers, the overall pattern suggests varying degrees of hyperinsulinemia across intervention groups. Similarly, TyG index—a surrogate marker for IR—increased in proportion to dietary fat content, with significant elevations observed in the M60 and M45 cohorts (*P* < 0.05; **Figures 2A–C**).

Dyslipidemia was evident in all intervention groups, characterized by significantly increased serum TG, TC, and LDL-C levels compared to the CON group (**Figures 2D–G**). The magnitude of lipid elevation correlated positively with dietary fat percentage (M60 > M45 > M10). Although HDL-C levels also rose with increasing fat intake, this paradoxical elevation is consistent with high-fat feeding models and may not signify improved cardiovascular protection in the context of broader metabolic dysfunction.

Dietary intervention disrupted adipokine homeostasis and provoked systemic inflammation. Adiponectin levels inversely correlated with dietary fat content, decreasing significantly from the CON baseline to the M60 group. Conversely, leptin levels followed a dose-dependent increase. Pro-inflammatory cytokines (TNF-α, IL-1β, IL-6) were elevated across all model groups (M60 > M45 > M10), confirming that diets with higher fat-to-carbohydrate ratios exacerbate chronic low-grade inflammation and adipocyte dysfunction (**Figures 2H–L**).

### 3.3 A higher fat diet differentially induces lipid deposition, predominantly in the liver of mice

**Figure 3:**
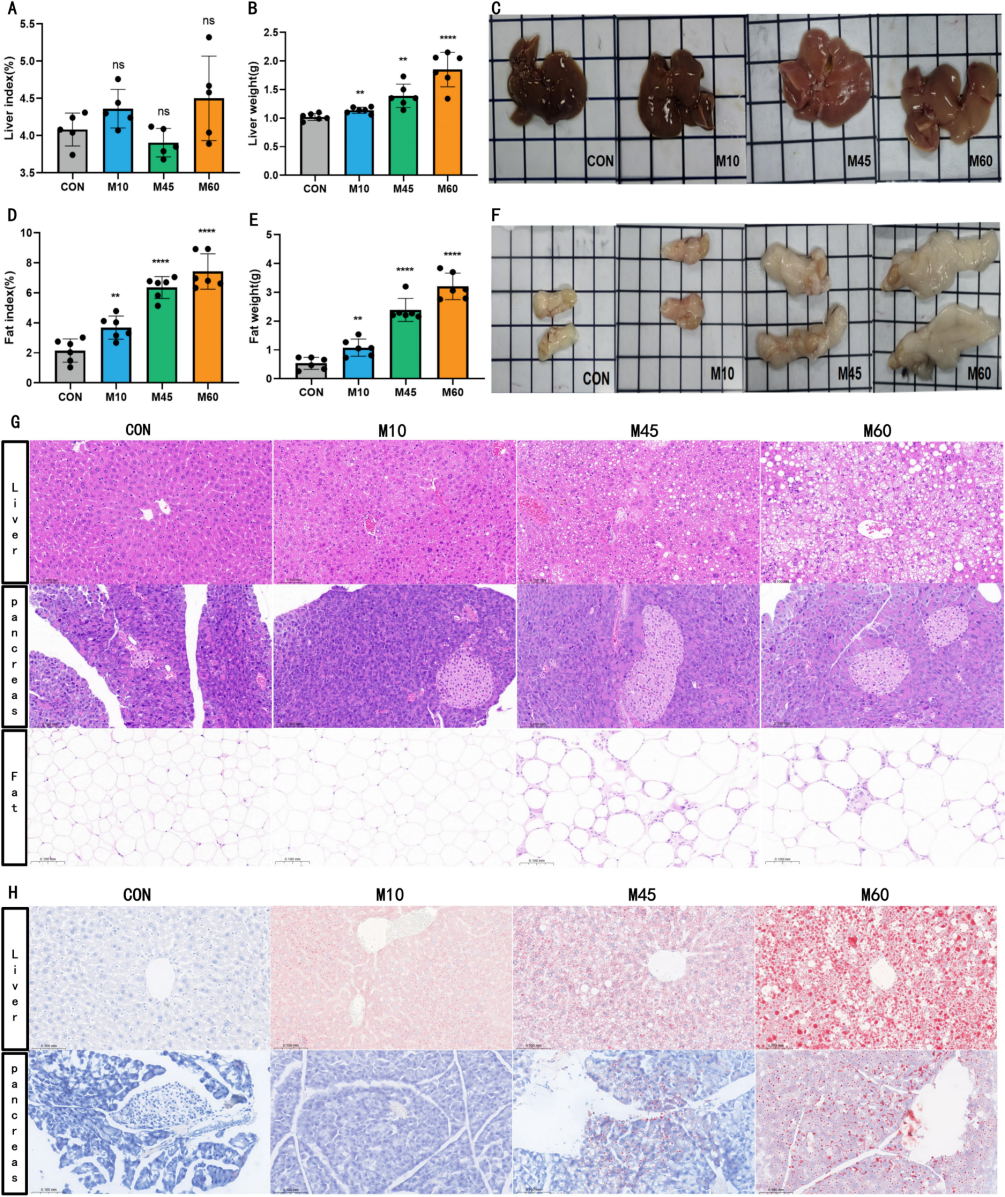
Morphological characteristics of tissues and organs in different carbohydrate-fat ratio diets. **(A)** Liver index of mice in each group. **(B)** Liver wet weight of mice in each group. **(C)** Representative liver images of mice in each group. **(D)** Fat index of mice in each group. **(E)** Fat wet weight of mice in each group. **(F)** Representative gross fat images of mice in each group. **(G)** H&E of liver、pancreas and adipose tissue (40×, scale bar: 100 μm). **(H)** Oil red staining of liver and pancreas (40×, scale bar: 100 μm). Data are expressed as mean ± SD, n = 6 for all data points. In statistical analysis, all values are expressed as mean ± SD. * indicates statistical difference between the model and control groups, ^*^P < 0.05, ^**^P < 0.01, ^***^P < 0.001.

High-fat feeding induced significant hepatomegaly and visceral adiposity. Liver wet weight increased in proportion to dietary fat content (*P* < 0.05), suggesting compensatory hypertrophy or hyperplasia in response to lipid overload. Gross examination revealed that while CON livers maintained a smooth, reddish-brown appearance with normal elasticity, experimental groups exhibited progressive pallor and a granular, steatotic texture—most notably in the M60 group. Similarly, visceral fat accumulation and the fat index were significantly elevated in a dose-dependent manner (**Figures 3A–F**).

H&E staining confirmed the macroscopic findings. Control livers displayed intact lobular architecture without lipid deposition. In contrast, the M60, M45, and M10 groups exhibited varying degrees of hepatic steatosis, hepatocyte ballooning, and necrosis. Adipose tissue analysis revealed a gradient of inflammatory cell infiltration (M60 > M45 > M10 > CON), indicating profound adipocyte dysfunction. Pancreatic tissue showed only mild, non-significant lesions across all groups (**Figure 3G**). Oil Red O staining further quantified the lipid burden: hepatic lipid accumulation was dose-dependent and markedly more severe than pancreatic deposition, identifying the liver as the primary site of ectopic fat storage in this model (**Figure 3H**).

### 3.4 Proteomic profiling identifies hepatic proteins and pathways activated by a higher fat diet in mice

**Figure 4:**
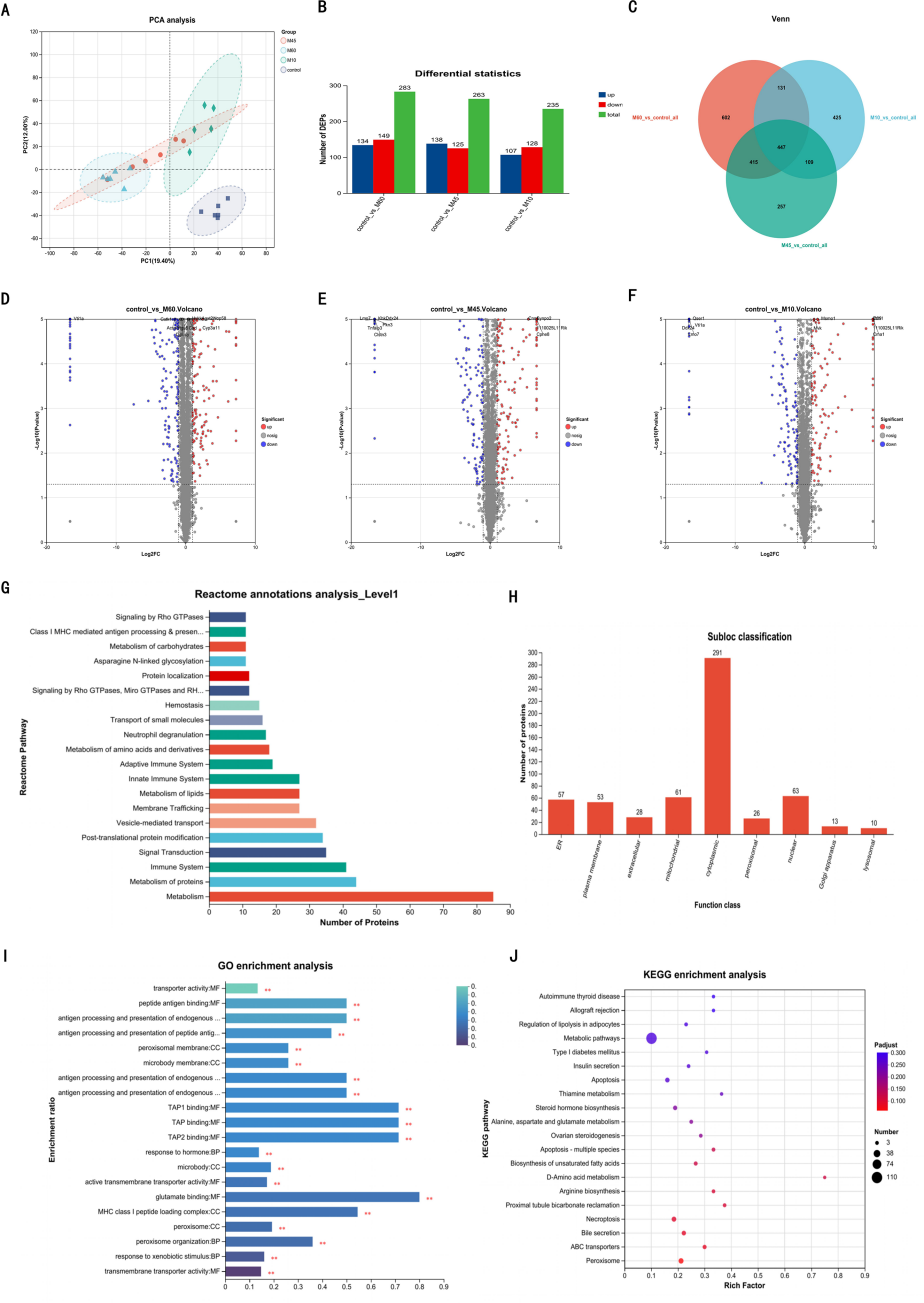
Proteomics analysis. **(A)** PCA analysis diagram. **(B)** Bar chart of DEPs in each model group. **(C)** Venn diagram of DEPs in each model group. **(D-F)** Volcano plot of the DEPs in each model group. **(G)** Reactome annotations analysis of the DEPs. **(H)** Subcellular distribution for DEPs. **(I)** GO analysis for DEPs. **(J)** KEGG pathway annotation for DEPs.

Based on the pathologic findings (Sections 3.1–3.3) demonstrating that the liver is the primary target organ for diet-induced metabolic dysfunction—particularly the rapid onset of obesity and T2DM in the M60 group—hepatic tissues were subjected to high-throughput proteomic analysis. The objective was to identify molecular signatures specific to the high-fat phenotype and explore the underlying pathogenic pathways. Principal Component Analysis (PCA) revealed tight clustering within replicates, indicating high reproducibility. Notably, the M45 group clustered intermediately between the M10 and M60 cohorts, while the CON group formed a distinct cluster separated from all model groups, mirroring the graded phenotypic severity observed *in vivo* (**Figure 4A**).

DEPs were defined by strict criteria (FC > 2, *P* < 0.05). Comparative analysis revealed a core set of 447 proteins modulated in all intervention groups. However, to isolate the specific factors contributing to the accelerated pathology in the M60 cohort, we focused on the 602 DEPs unique to this group (**Figures 4D–F**). This strategy allowed for the targeted exploration of biological pathways exclusively activated by high-fat intake, independent of the moderate changes seen in the M10 and M45 groups.

Reactome pathway analysis of the specific DEPs in the M60 group identified metabolism as the most enriched category, followed by lipid metabolism, protein metabolism, immune system, and signal transduction (**Figure 4G**). Subcellular localization prediction revealed that these unique DEPs were predominantly localized to the cytoplasm, nucleus, and mitochondria, consistent with their metabolic and regulatory roles (**Figure 4H**). GO analysis highlighted key functional clusters among the top 20 enriched terms (**Figure 4I**). Biological processes (BP) were centered on responses to foreign stimuli, peroxisome organization, hormone response, and antigen processing. Cellular components (CC) were enriched in peroxisomal and microbody structures, as well as the MHC class I peptide loading complex. Molecular functions (MF) primarily involved transporter activity and peptide antigen binding. Furthermore, KEGG pathway analysis (**Figure 4J**) identified significant enrichment in broad metabolic pathways, including unsaturated fatty acid biosynthesis, bile secretion, steroid hormone synthesis, and thiamine metabolism. Critical regulatory pathways related to cell death (apoptosis, necroptosis), endocrine function (insulin secretion, regulation of lipolysis), and diabetes-associated signaling (Type 1 diabetes mellitus) were also prominently represented.

### 3.5 Metabolomic profiling identifies hepatic metabolites and pathways activated by a higher fat diet in mice

Metabolomics focuses on the quantitative profiling of dynamic metabolite fluctuations across biological hierarchies (cells to biological systems) to systematically elucidate the biochemical regulatory networks within the organism (26). The advancement of high-throughput technologies has enabled researchers to rapidly analyze circulating metabolites on a large scale. By simultaneously conducting qualitative and quantitative analyses of low-molecular-weight species—such as sugars, organic acids, lipids, amino acids, and aromatic hydrocarbons—specific differential targets can be identified. T2DM is particularly suitable for such metabolomic interrogation because it is a complex disease driven by multiple metabolic factors and influenced by physiological changes like diet and physical exercise (27). In this study, we utilized liver organ samples from mice to observe metabolic characteristics under dietary intervention, aiming to identify group-specific differential metabolites. Specifically, metabolites unique to the M60 group were subjected to bioinformatics analysis to explore biological pathways that may promote the occurrence of T2DM.

**Figure 5:**
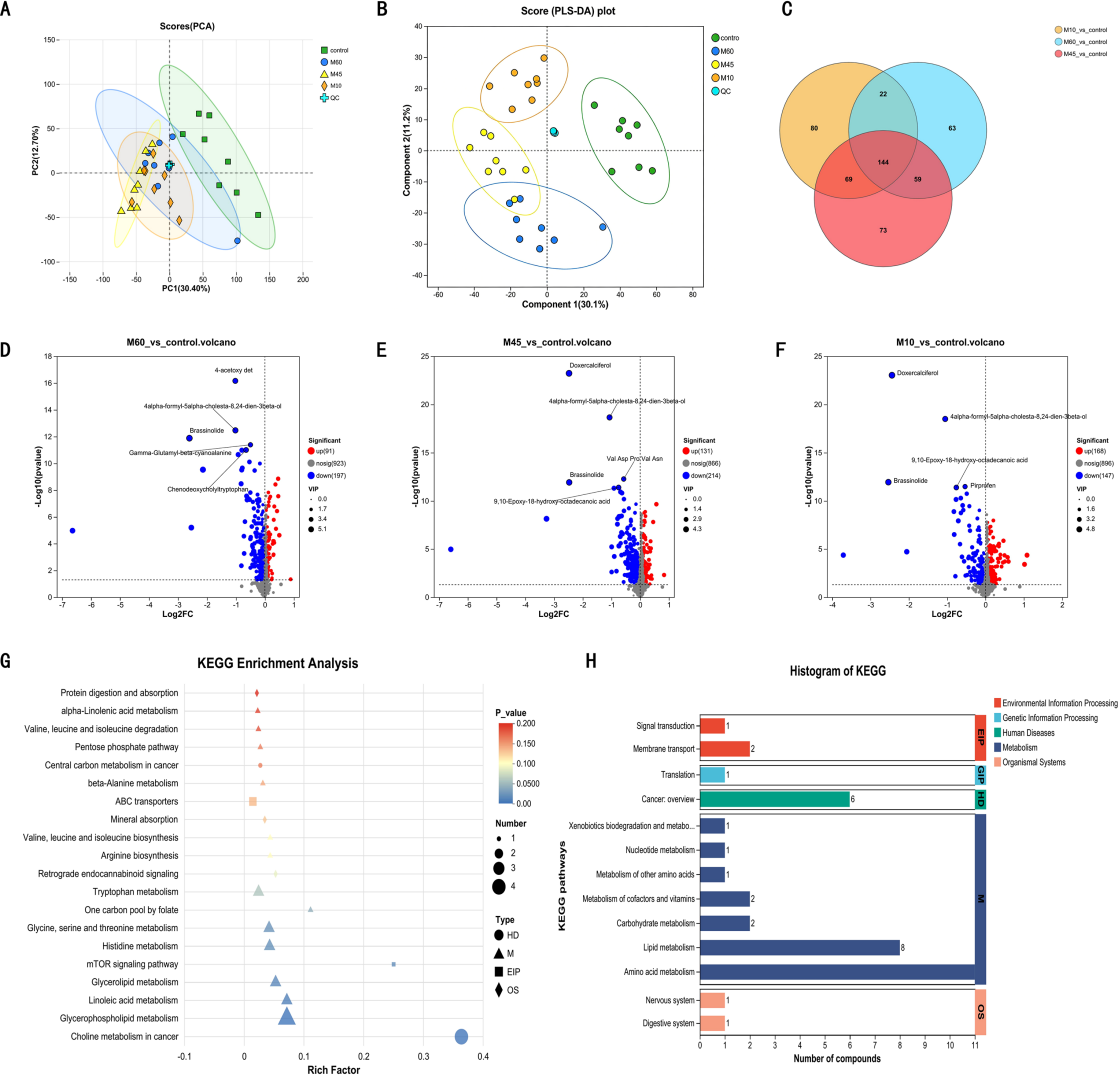
Metabolomics analysis. **(A)** PCA analysis diagram. **(B)** PLS-DA analysis diagram. **(C)** Venn diagram of DEMs in each model group. **(D-F)** Volcano plot of the DEMs in each model group. **(G)** KEGG Enrichment analysis of the DEMs. **(H)** Histogram KEGG of the DEMs.

PCA revealed that the CON group was distinctly separated from the three model groups. Although the model groups exhibited some intersection, a clear trend of separation was observable, and the variation degrees between and within each group were robust (**Figure 5A**). To identify common and unique metabolic differences, a Venn analysis was performed. As illustrated in the Venn diagram (**Figure 5C**), the intersection of differential metabolites among the three model groups was analyzed. It was found that 144 differential metabolites were common to the M60, M45, and M10 groups, while 63 metabolites were unique to the M60 group. Consequently, we designated these 63 unique metabolites as the target set for subsequent analysis.

Differentially expressed metabolites (DEMs) were identified using Variable Importance in the Projection (VIP) values from OPLS-DA (or PLS-DA in cases of OPLS-DA overfitting), combined with FC and p-values from univariate analysis. The screening criteria were established as follows: (1) OPLS-DA VIP ≥ 1, representing the discriminatory contribution of the metabolite to group classification; (2) FC > 1, indicating a quantitative divergence between the control and experimental groups; and (3) *P* < 0.05. Using these thresholds, a total of 4276 liver metabolites were profiled. Within the M60 group, 288 DEMs were detected, comprising 91 upregulated and 197 downregulated species. The top five DEMs—Brassinolide, Chenodeoxycholyltryptophan, Gamma-Glutamyl-beta-cyanoalanine, 4-acetoxy det, and 4alpha-formy-5alpha-cholesta-8,24-dien-3beta-ol—are highlighted in the results (**Figures 5D-F**).

Subsequently, KEGG enrichment and functional pathway analyses were performed on the differential metabolic sets. The primary enriched pathways included glycerophospholipid metabolism, linoleic acid metabolism, glycerol ester metabolism, mechanistic Target Of Rapamycin Kinase (mTOR) signaling pathway, choline metabolism in cancer, histidine metabolism, metabolism of glycine, serine, and threonine, tryptophan metabolism, arginine biosynthesis, biosynthesis of valine, leucine, and isoleucine, β-alanine metabolism, pentose phosphate pathway, degradation of valine, leucine, and isoleucine, α-linolenic acid metabolism, and protein digestion and absorption. According to KEGG topological analysis, the top five ranked pathways were glycerophospholipid metabolism, histidine metabolism, glycerol ester metabolism, tryptophan metabolism, and cysteine and methionine metabolism (**Figure 5G**). Identifying and characterizing these metabolic alterations is central to our analysis, as key metabolites are typically linked to functional shifts. Our analysis of KEGG functional pathways revealed that HFD feeding disrupted liver processes involving lipid, amino acid, carbohydrate, nucleotide, and cofactor/vitamin metabolism, thereby impacting the digestive and nervous systems. Concurrently, environmental information processing mechanisms, such as signal transduction and transmembrane transport, were also found to be disordered (**Figure 5H**).

### 3.6 Integrated proteomics and metabolomics reveal pathways related to higher fat diet induced T2DM

Initial screening of the differential datasets identified 282 DEPs and 32 DEMs. This disparity suggests a systemic response pattern where HFD induces widespread proteomic alterations while metabolic changes remain more concentrated. To investigate the interplay between these omics layers, we employed an O2PLS model to project the datasets into a joint subspace. The resulting loadings plot revealed a distinct distribution: whereas the majority of proteins (blue) clustered near the origin, metabolites (green) and a specific subset of proteins were dispersed peripherally. This configuration indicates that the metabolic alterations did not occur in isolation but were tightly coupled with specific proteomic shifts.

**Figure 6:**
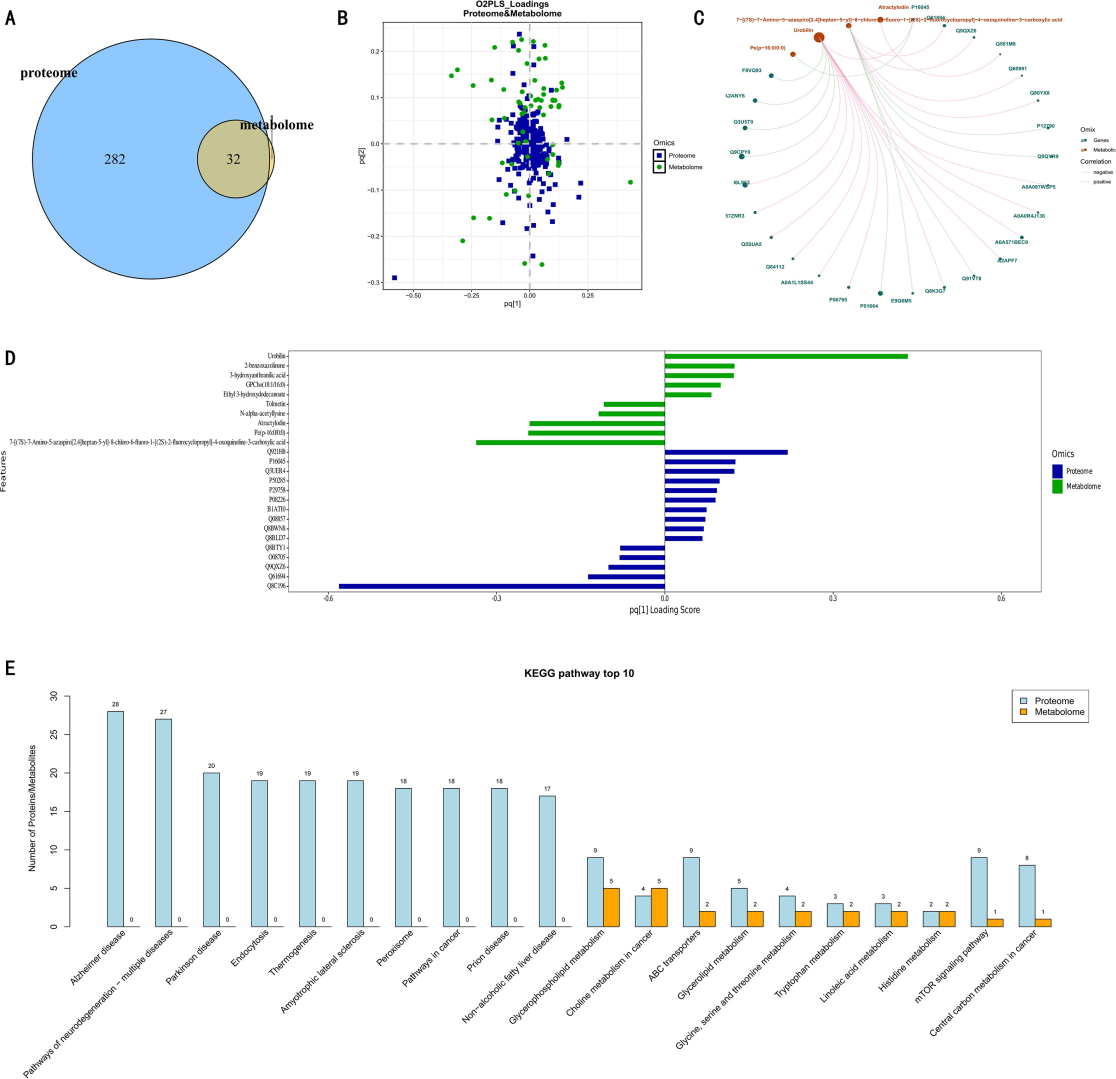
Integrated multi-omics analysis. **(A)** Venn diagram of DEMs and DEPs. **(B)** O2PLS model loadings plot of proteome and metabolome. **(C)** Differential protein/metabolite correlation network diagram. **(D)** Differential protein and differential metabolite load column charts. **(E)** Top 10 pathways with the highest differences in proteins/metabolites.

Quantitative ranking of the O2PLS model features (**Figure 6D**) listed the top 15–20 contributors to the first joint principal component (pq (1)). Notably, urobilin and several fatty acid derivatives—specifically GPCho (18:1/20:4) and Ethyl 3-hydroxydodecanoate—were identified as the primary metabolic drivers. Concurrently, specific proteins (e.g., Q92…) were highlighted for their strong association with these metabolic shifts. To visualize these interactions, a chord diagram/correlation network was constructed linking metabolites (orange/brown nodes) to proteins (teal nodes), with pink and gray lines representing positive and negative correlations, respectively. Within this network, key lipids such as Pe(p - 16:0/9:0) (a phospholipid) and GPCho (18:1/20:4) (containing fatty acyl chains) were prominent. The association between these lipids and specific protein expression suggests that the disorder of lipid metabolism is closely linked to these proteomic changes. Furthermore, joint KEGG pathway enrichment analysis revealed co-enrichment in “Glycerophospholipid metabolism” and “ABC transporters.” Collectively, these findings demonstrate that HFD disrupts systemic homeostasis through coordinated perturbations of proteins and metabolites within specific lipid metabolic pathways.

### 3.7 The mechanistic study on the effects of diets with different glucose-lipid ratios on T2DM onset in mice

Previous studies have characterized the impact of diets with varying sugar-lipid ratios on T2DM pathology, while high-throughput proteomics and metabolomics have delineated the pathways driving disease onset. Our results indicated that differential metabolites were significantly enriched in lipid metabolism, with lipids and lipid-like molecules dominating the metabolic reprogramming induced by high-fat diets. Concurrently, differential proteins were intimately linked to these lipid pathways, showing subcellular localization patterns that suggest a mitochondria-centered, multi-organelle coordination of lipid homeostasis. Integrating these phenotypic and omics data, we validated key targets within mitochondrial function and lipid regulation. This confirms that the coupled pathway of mitochondrial impairment and lipid metabolism disorder constitutes a distinct biological network mechanism by which high-fat ratio diets accelerate T2DM pathogenesis. Consequently, future intervention strategies should simultaneously target mitochondrial integrity and lipid homeostasis to effectively prevent T2DM.

**Figure 7:**
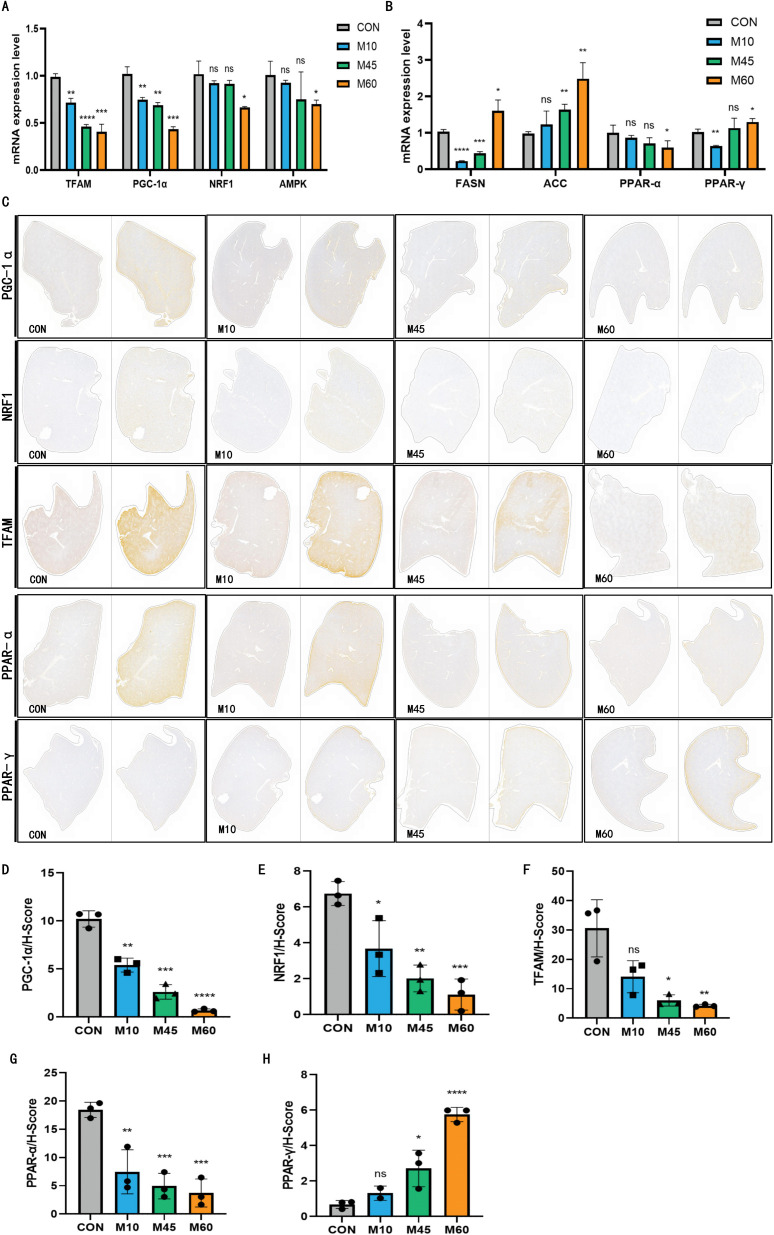
The expression of mitochondrial function and lipid metabolism homeostasis-related molecular proteins and RNAs in different sugar-fat ratio diet groups. **(A, B)** The mRNA levels of key mitochondrial function and lipid metabolism genes. **(C)** Immunohistochemical pathological images in liver tissue. **(D-H)** Immunohistochemical positive area H-Scores in liver tissue. * Compared with the CON group, ^*^P < 0.05, ^**^P < 0.01, ^***^P < 0.001,^****^ *P* < 0.0001. H-score: It refers to Histochemistry score (tissue chemical score), the larger the value, the stronger the comprehensive positive intensity in terms of both the depth and quantity of positivity.

To corroborate these findings at the transcriptional level, qRT-PCR was employed to quantify mitochondrial biogenesis-related mRNA expression in liver tissues. The results (**Figure 7A**) demonstrated that expression levels of TFAM, PGC-1α, NRF1, and AMPK were significantly lower in the M10, M45, and M60 groups compared to the CON group. Notably, this downregulation followed a dose-dependent trend, intensifying as dietary fat content increased (from M10 to M60). Specifically, the M60 treatment significantly suppressed mitochondrial biogenesis genes (*P* < 0.05). Immunohistochemistry further verified the pivotal role of these mitochondrial metabolic proteins. Consistent with the mRNA data, protein expression levels of PGC-1α, NRF1, and TFAM were reduced in all model groups compared to controls, evidenced by diminished brown-yellow staining intensity and decreased H-Scores. Furthermore, this reduction in protein expression progressively deepened with increasing dietary fat (from M10 to M45 to M60) (**Figure 7C**), confirming the suppression of mitochondrial biogenesis under high-fat conditions.

**Figure 7B** illustrates the mRNA expression profiles of key molecules involved in fatty acid metabolism (FASN, ACC) and nuclear receptors (PPAR-α/γ) across different diet groups (CON, M10, M45, M60). FASN expression exhibited divergent patterns: it was significantly upregulated in the M60 group compared to the CON group, whereas the M10 and M45 groups displayed a decreasing trend. Conversely, ACC expression followed a progressive upward trajectory, increasing in parallel with dietary fat content. regarding the nuclear receptors, PPAR-α expression showed a consistent downward trend in all model groups, with the M60 group being significantly lower than the CON group. The expression of PPAR-γ was more complex: it significantly decreased in the M10 group but shifted to an increasing trend in the M45 group, culminating in a significant increase in the M60 group. To corroborate these transcriptional changes, immunohistochemistry was employed to evaluate the protein expression of PPAR-α and PPAR-γ in mice fed diets with varying sugar-lipid ratios. The results indicate that PPAR-α protein levels progressively declined from the M10 group through the M45 group to the M60 group, compared with the CON group. In contrast, PPAR-γ protein expression followed the opposite pattern, reaching its peak in the M60 group. Notably, the difference in PPAR-γ expression between the M45 and M60 groups was statistically significant (**Figure 7**).

## 4 Discussion

In constructing the experimental model, the C57BL/6J mouse strain was selected due to its well-documented susceptibility to diet-induced obesity. While other common strains, such as Kunming, ICR, and BALB/c mice, exhibit similar responses to HFD (28), the C57BL/6J remains the standard for metabolic research. Conversely, the ob/ob mouse was excluded because its obesity stems from a leptin gene mutation causing hyperphagia, which fails to recapitulate the natural pathophysiological progression from normal glucose tolerance to hyperglycemia driven by a sugar-fat diet (29).The rationale for our dietary design was twofold, integrating rigorous preliminary screening with established model standardization. Initially, a pilot study screened a gradient of five energy densities (ranging from 10% to 70% fat) to map the phenotypic response to varying sugar-lipid ratios. Informed by these preliminary findings, we strategically focused on three pivotal ratios aligned with standardized formulations from Research Diets. Specifically, the 60% fat diet was selected to represent the classic lipotoxicity-driven HFD model, while the 45% fat diet served as the standard High-Fat High-Sugar (Western Diet) model. This approach facilitates a comprehensive analysis of how diet structure continuously influences metabolic phenotypes and drives specific molecular mechanisms, including the mitochondrial lipid remodeling observed in our omics profiles.

Intervention with varying sugar-lipid diets revealed that dysglycemia significantly lags behind the obesity phenotype. Specifically, while weight differentiation initiated early (week 2 in the high-fat M60 group), blood glucose divergence was not observable until week 6. Distinct from traditional HFD models, we systematically characterized the temporal onset of metabolic disorders. The 12-week endpoint was established based on the M60 group, where T2DM incidence reached 90%. At this juncture, the M45 group primarily exhibited Impaired Glucose Tolerance (only 30% T2DM incidence), while the M10 group maintained normal glucose levels, implying that a dietary fat contribution exceeding 45% serves as a critical threshold for FBG disruption. Weight trajectories further corroborated a time-dependent acceleration: significant divergence from the CON group occurred at week 2 for M60 (P < 0.01), but was delayed to weeks 6 and 8 for the M45 and M10 groups, respectively. Collectively, these data establish that the dietary fat ratio governs both the magnitude (dose-dependence) and the velocity (temporal acceleration) of obesity and T2DM pathogenesis.

Following the sugar-lipid diet intervention, the lipid profiles in all model groups exhibited marked alterations, characterized by universal increases in TG, TC, LDL-C, and HDL-C. Although HDL-C is conventionally regarded as “good cholesterol,” its elevation here likely reflects the increased intake of saturated fatty acids (30) and disease-associated remodeling. Pathological states such as T2DM are known to alter the proteomic and lipidomic composition of HDL particles, thereby compromising their functional integrity (31). Consistent with the role of IR as a common mechanism underlying obesity and T2DM, the HOMA-IR and TyG indices—established surrogate markers for IR (32)—demonstrated clear trends: HOMA-IR increased stepwise with dietary fat content, while the TyG index correlated positively with the fat energy ratio. These findings corroborate the central role of lipotoxicity in expanding IR (33). Concurrently, adipokine dysregulation was evident. Adiponectin levels declined in all model groups, potentially weakening its stimulation of fatty acid oxidation and exacerbating ectopic lipid deposition—a key driver of T2DM risk (34, 35). Conversely, leptin, which normally regulates weight by inhibiting orexigenic and stimulating anorexigenic pathways (36), rose dramatically in the M60 group to 1898 ng/mL (vs. 691.3 ng/mL in CON), signaling the onset of leptin resistance typified by hyperleptinemia (37). Furthermore, the diet triggered a systemic pro-inflammatory cascade, evidenced by elevated TNF-α, IL-1β, and IL-6 levels. However, unlike the linear metabolic shifts, inflammation did not exhibit strict dose-dependence on the fat energy ratio; while the M60 group showed the highest expression, the M10 and M45 groups were comparable, suggesting that even moderate sugar-lipid distinct ratios suffice to activate inflammatory signaling.

As the central hub of metabolism, the liver exhibited high sensitivity to lipid overload in this study. H&E staining revealed a gradient of pathological changes, including hepatic steatosis, hepatocellular necrosis, ballooning, and inflammatory cell infiltration, which intensified in a dose-dependent manner (M60 > M45 > M10). Correspondingly, both the area and intensity of lipid staining increased in parallel with the dietary fat energy ratio. in contrast, adipose tissue presented primarily with macrophage infiltration without severe structural collapse, while pancreatic tissue in all model groups remained largely free of severe lesions indistinguishable from the CON group. These observations indicate that the liver was the most profoundly affected organ under different sugar-lipid ratio diets. This susceptibility is likely attributable to anatomical and physiological factors. Anatomically, unlike skeletal muscle (which mediates glucose uptake) or adipose tissue (which stores lipids), the liver directly receives nutrient-rich blood from the intestine via the portal vein, thereby exposing it to high postprandial concentrations of free fatty acids (FFA) and glucose earlier than peripheral organs (38). Furthermore, the liver plays a critical regulatory role in energy balance; when processing excessive substrates, it may release signaling molecules such as FGF21, which can induce lipolysis in adipose tissue and paradoxically amplify lipotoxicity (39). Finally, according to the spillover hypothesis, when dietary fat exceeds the storage threshold of adipose tissue, lipids overflow into the liver, triggering non-alcoholic fatty liver disease, whereas other organs like the pancreas may only succumb to lipotoxicity at later stages (40). Collectively, these mechanisms render the liver uniquely vulnerable to the early onset of lesions under sugar-lipid dietary interventions.

Building upon phenotypic observations of mice fed distinct sugar-lipid ratios, this study characterized the proteomic landscape specific to the M60 high-fat diet group. Following the exclusion of proteins shared with the M10, M45, and CON groups, the unique M60 proteomic signature was isolated for functional interrogation. Integrated GO annotation and enrichment analyses revealed that these target proteins are primarily implicated in the transmembrane transport of metabolites and ions. Notably, the enrichment of ATP-dependent activities (e.g., ATPase) points to altered energy metabolism; this compromised ATP synthesis likely disrupts ion pump efficacy, precipitating calcium or FFA accumulation and subsequent mitochondrial dysfunction (41). Corroborating this, KEGG and Reactome pathway analyses highlighted a robust enrichment in metabolic regulation, specifically lipid metabolism (including unsaturated fatty acid biosynthesis, adipocyte lipolysis, and steroid hormone synthesis), amino acid metabolism (alanine, aspartate, and glutamate), and cofactor/vitamin processing (such as thiamine and retinol).The proteomic data further suggests a mechanism of mitochondrial stress. Excessive fatty acid influx into mitochondria for oxidation is known to generate elevated reactive oxygen species, causing cytochrome c release and apoptosis (42). Furthermore, the physical contact between mitochondria and lipid droplets is critical for fatty acid transfer and β-oxidation. In high-fat environments, the accumulation of lipid metabolites has been reported to induce K243-specific acetylation and ubiquitin-proteasome degradation of Mfn2; this disruption of the Mfn2-Hsc70 mediated contact blocks fatty acid oxidation, thereby triggering lipotoxicity (43). Subcellular localization analysis indicated that the differentially expressed proteins in the M60 group are predominantly resident in the mitochondria, endoplasmic reticulum (ER), and plasma membrane. Mitochondria serve as central hubs for energy conversion, fatty acid oxidation, and calcium homeostasis, proving essential for organelle crosstalk. In particular, the mitochondria-ER interface acts as a critical node for lipid synthesis and calcium balance, an interaction frequently altered in obesity and metabolic insulin resistance across tissues such as the liver and muscle (44). Collectively, these proteomic characteristics underscore a core, mitochondria-centered coordination of multicellular organelles in the regulation of lipid metabolism.

Integrated metabolomic and bioinformatic profiling revealed distinct hepatic signatures in mice fed different sugar-lipid ratio diets compared to controls. The metabolic variance was chiefly driven by Brassinolide, 4α-formyl-5α-cholesta-8,24-dien-3β-ol, and Doxercalciferol. Brassinolide, a brassinosteroid phytohormone, was notably identified; known for regulating plant sugar metabolism and photosynthetic accumulation (45, 46), its presence here correlates with fatty acid accumulation and associated gene expression profiles (47). Similarly, the detection of 4α-formyl-5α-cholesta-8,24-dien-3β-ol points to perturbed cholesterol biosynthesis. Structurally resembling a sterol intermediate, this 4α-formyl moiety likely represents an incompletely demethylated by-product or a tissue-specific metabolite (48, 49), potentially enhancing nuclear receptor binding to modulate lipid or inflammatory responses. Furthermore, Doxercalciferol (1α-hydroxyvitamin D_2_), a synthetic steroid derivative and vitamin D receptor agonist, was significantly altered; its activity relies on hepatic hydroxylation and is implicated in the regulation of renal lipid metabolism (50).At the network level, KEGG enrichment analysis of the M60 group highlighted profound shifts in lipid and amino acid metabolism. The co-enrichment of glycerophospholipid and glycerolipid pathways suggests extensive remodeling of liver cell membrane phospholipids (51). As glycerophospholipids are critical for mitochondrial membrane integrity, their metabolic disruption directly compromises the electron transport chain (52). Concurrently, the alignment of histidine/aspartate metabolism with the mTOR signaling pathway reflects a breakdown in amino acid sensing. Dysregulated mTORC1 signaling may drive indiscriminate protein and lipid synthesis via S6K and 4E-BP phosphorylation while inhibiting mitophagy; this failure to clear damaged mitochondria likely exacerbates the metabolic burden imposed by the high-fat/high-sugar regimen (53).

Integrated proteomic and metabolomic profiling unveiled a synchronized dysregulation of the hepatic landscape under HFD conditions. This multi-omics analysis demonstrates that dietary lipid overload not only elicits extensive proteome reprogramming but also precipitates specific lipidomic imbalances, preferentially targeting glycerophospholipid metabolism and ABC transporter pathways. Corroborated by subcellular localization data, these perturbations appear centrally driven by mitochondrial dysfunction. Specifically, we observed marked alterations in fatty acid derivatives, including Pe(p-16:0/9:0) and GPCho (18:1/20:4). As Phosphatidylethanolamine (PE) is critical for negative membrane curvature and cristae packing, the observed shifts in PE composition imply a compromise in mitochondrial structural integrity (54). Concurrently, changes in GPCho (18:1/20:4)—a phosphatidylcholine species enriched in arachidonic acid (20:4)—contribute to a disrupted PC/PE ratio, a recognized hallmark of mitochondrial failure associated with obesity and diabetes (55). Furthermore, metabolic stalling was evidenced by the accumulation of ethyl 3-hydroxydodecanoate. The buildup of this 3-hydroxy fatty acid derivative signals a bottleneck in the β-oxidation flux, where substrate overload exceeds mitochondrial oxidative capacity, preventing the complete thiolysis of intermediates (56). Collectively, these omics signatures suggest that mitochondrial dysfunction, characterized by structural fragility and oxidative inefficiency, drives lipotoxicity and oxidative stress. This loss of cellular metabolic flexibility provides a molecular basis for the onset of insulin resistance and the progression of T2DM.

This study systematically delineates the impact of dietary sugar-lipid ratios on T2DM progression, identifying a critical breakdown in the mitochondrial function-lipid metabolism regulatory network. Our data reveal that high-fat diets—particularly in the M60 group (>60% fat)—significantly accelerate metabolic dysregulation by suppressing the AMPK-PGC-1α signaling axis. Specifically, we observed a universal downregulation of mitochondrial quality control genes (AMPK, PGC-1α, TFAM, NRF1) across high-fat groups. As a master energy sensor (57, 58), AMPK normally phosphorylates PGC-1α at Ser568 to initiate transcriptional competence (59). The suppression of this axis compromises PGC-1α, a central orchestrator of mitochondrial biogenesis and function in high-energy tissues (60). Consequently, the downstream activation of NRF1/2 and TFAM is blunted, impairing mitochondrial DNA replication and quality control (61, 62). This transcriptional silencing mirrors the diminished hepatic PGC-1α levels observed in non-alcoholic fatty liver disease and T2DM patients, linking mitochondrial decline to IR (64). Furthermore, the impairment of PGC-1α directly destabilizes the lipid metabolic network by failing to co-activate PPARα (63). Our results demonstrate a consistent imbalance in the PPAR-γ/α axis across all high-fat cohorts, characterized by elevated PPAR-γ and suppressed PPAR-α levels. This molecular shift suggests a dual failure: downregulated PPAR-α reduces mitochondrial β-oxidation capacity, while aberrant PPAR-γ activation promotes adipogenesis over lipolysis (65, 66). The resulting lipid overload interacts with insulin signaling pathways, precipitating lipotoxicity and systemic IR (67). In turn, IR exacerbates free fatty acid release, accumulating toxic metabolites (68) and perpetuating a deleterious feedback loop of metabolic deterioration.

We acknowledge several important limitations in this current study. First, while statistical analysis conclusively established no significant differences in average food intake among the dietary groups throughout the core intervention period—effectively excluding differential caloric consumption driven by diet palatability as a major confounding factor in our metabolic phenotyping—we recognize the recording frequency proved insufficient to support a detailed temporal graphical presentation within the Results section. In follow-up studies, we will implement significantly more frequent and systematic food intake monitoring to enable comprehensive longitudinal assessment. Second, water intake represents a significant omission in the current study. We plan to incorporate routine water intake monitoring in subsequent experiments to fully account for this potential confounder. Third, the omics-derived pathways reported here represent only initial exploratory insights with preliminary experimental validation; the precise underlying mechanisms remain to be definitively elucidated. We are committed to conducting dedicated mechanistic investigations in future studies to robustly validate and significantly extend these observations.

## 5 Conclusion

In summary, a HFD exerts more pronounced adverse effects compared to other macronutrient compositions, accelerating T2DM through a dual interaction network involving mitochondrial dysfunction and lipid metabolic reprogramming, which collectively disrupt systemic metabolic homeostasis.

## Data Availability

The original contributions presented in the study are included in the article/**Supplementary Material**. Further inquiries can be directed to the corresponding authors.
