# Supplementary material for: Effects of different sugar-lipid ratio diets on the occurrence of type 2 diabetes mellitus
Source: Front Endocrinol (Lausanne). 2026 Apr 23;17:1768263. doi: 10.3389/fendo.2026.1768263 (PMC13149151; doi:10.3389/fendo.2026.1768263)
Supplement: Supplementary file 1 [file DataSheet1.docx]

**Supplementary materials**

**Table S1 The dietary components of each dietary group**

| **Types of diet** | 60%F20%C diet | | 45%F35%C diet | | 10%F70%C diet | | Normal diet | |
| --- | --- | --- | --- | --- | --- | --- | --- | --- |
|  | gm% | Kcal% | gm% | Kcal% | gm% | Kcal% | gm% | Kcal% |
| Protein | 26.2 | 20 | 24 | 20 | 19.2 | 20 | - | 23 |
| Carbohydrate | 26.3 | 20 | 41 | 35 | 67.3 | 70 | - | 65 |
| Fat | 34.9 | 60 | 24 | 45 | 4.3 | 10 | - | 12 |
| Total |  | 100 |  | 100 |  | 100 |  | 100 |
| Kcal/gm | 5.24 |  | 4.73 |  | 3.85 |  | 3.40 |  |
| **Ingredients** | gm | Kcal | gm | Kcal | gm | Kcal |  | |
| Casein | 200 | 800 | 200 | 800 | 200 | 800 |  |  |
| Cystine, L | 3 | 12 | 3 | 12 | 3 | 12 |  |  |
| Corn starch | 0 | 0 | 72.8 | 291 | 315 | 1260 |  |  |
| Maltodextrin | 125 | 500 | 100 | 400 | 35 | 140 |  |  |
| Saccharose | 68.8 | 275.2 | 172.8 | 691 | 350 | 1400 |  |  |
| Cellulose | 50 | 0 | 50 | 0 | 50 | 0 |  |  |
| Soya Oil | 25 | 225 | 25 | 225 | 25 | 225 |  |  |
| Lard | 245 | 2205 | 177.5 | 1598 | 20 | 180 |  |  |
| Composite minerals | 10 | 0 | 10 | 0 | 10 | 0 |  |  |
| Calcium hydrogen phosphate | 13 | 0 | 13 | 0 | 13 | 0 |  |  |
| Calcium carbonate | 5.5 | 0 | 5.5 | 0 | 5.5 | 0 |  |  |
| Potassium Citrate | 16.5 | 0 | 16.5 | 0 | 16.5 | 0 |  |  |
| H20 | 10 | 240 | 10 | 40 | 10 | 40 |  | |
| Multi-Vitamins | 2 | 0 | 2 | 0 | 2 | 0 |  |  |
| Hydrocholine tartrate | 0.05 | 0 | 0.05 | 0 | 0.05 | 0 |  |  |
| Total | 773.85 | 4057 | 858.15 | 4057 | 1055.05 | 4057 |  |  |

**Table S2 Primer sequence**

| Gene | Primer Name | Primer sequence |
| --- | --- | --- |
| Pik3r1 | mPik3r1-F | AAGTTGGTCTTTTGACGAGAGG |
|  | mPik3r1-R | GAACAAAATGTCAAGGAGGCAC |
| IRS1 | mIRS1-F | GCCAAACGAATGCTATCTCTCT |
|  | mIRS1-R | GGTAGCAACATAGACCATGCGT |
| AKT1 | mAKT1-F2 | CCAGGGCTCAAGTGAGGTTG |
|  | mAKT1-R2 | GGCTCTCCTGTCACCAAGATTA |
| PPARγ | mPPARγ-F | AGCTGTCATTATTCTCAGTGGAGAC |
|  | mPPARγ-R | TGTCATCTTCTGGAGCACCTTG |
| PGC-1α | mPGC-1α-F | AACTGCAGATTTGATGGAGCTAC |
|  | mPGC-1α-R | CATGTAGAATTGGCAGGTGGA |
| PPARα | mPPARα-F | GTTTGACTGAACCATGACAGGAA |
|  | mPPARα-R | TGCCACCGTTCTTTGGTAATA |
| ACC | mACC-F | CAGACTGGCTTGAACTGAGATTG |
|  | mACC-R | AACGTGAGAAGAGTGCATTGGA |
| TFAM | mTfam-F1 | CCTGCCTGTCCTGTAACTCACT |
|  | mTfam-R1 | GAAGCCTGATGGTGGTGGTG |
| FASN | mFasn-F | ATCCCAGTCTGAGTGATTAGCCT |
|  | mFasn-R | ACTATAGAGATTGGGCAGGTCCT |
| NRF1 | mNRF1-F1 | CCCGTGTTCCTTTGTGGTGA |
|  | mNRF1-R1 | TGGAGAACAACACAGATTCCATG |
| AMPK | mAMPK-F2 | TGTTCATTGTGGGCTCTGACAT |
|  | mAMPK-R2 | TCTAAATCAGGTTACTCTGGGCAA |
| β-actin | β-actin-F2 | CCTAGCACCATGAAGATCAAGAT |
|  | β-actin-R2 | ACTCATCGTACTCCTGCTTGCT |

**Table S3 Protein Information Table**

| Peptide number | Protein number | Protein group number |
| --- | --- | --- |
| 89708 | 80901 | 7139 |


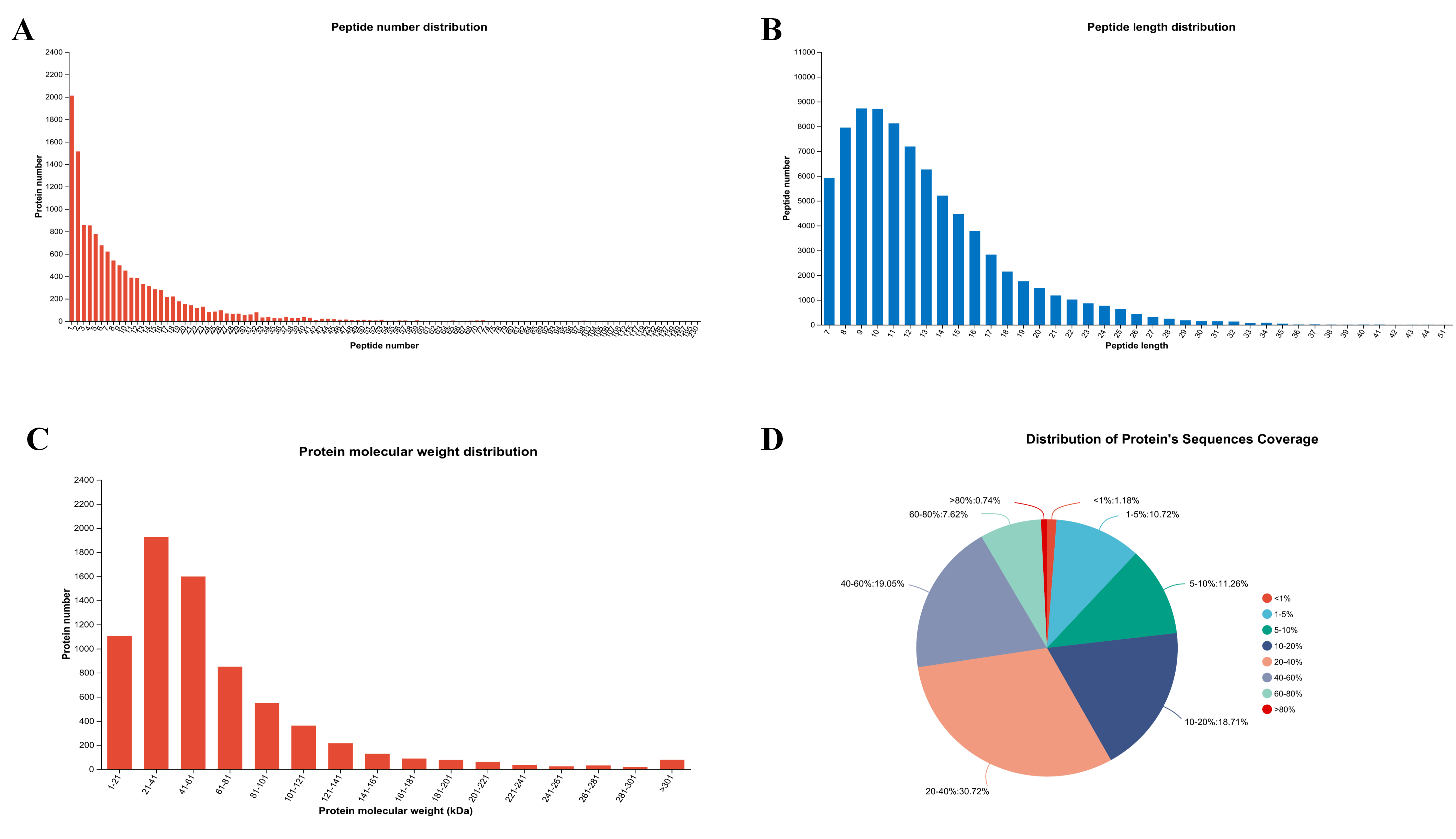


**Figure S1** Peptide Segment and Protein Information Graphs (A) Distribution map of peptide segment quantities. (B) Distribution map of peptide segment lengths. (C) Distribution map of protein molecular weights. (D) Distribution map of protein coverage.


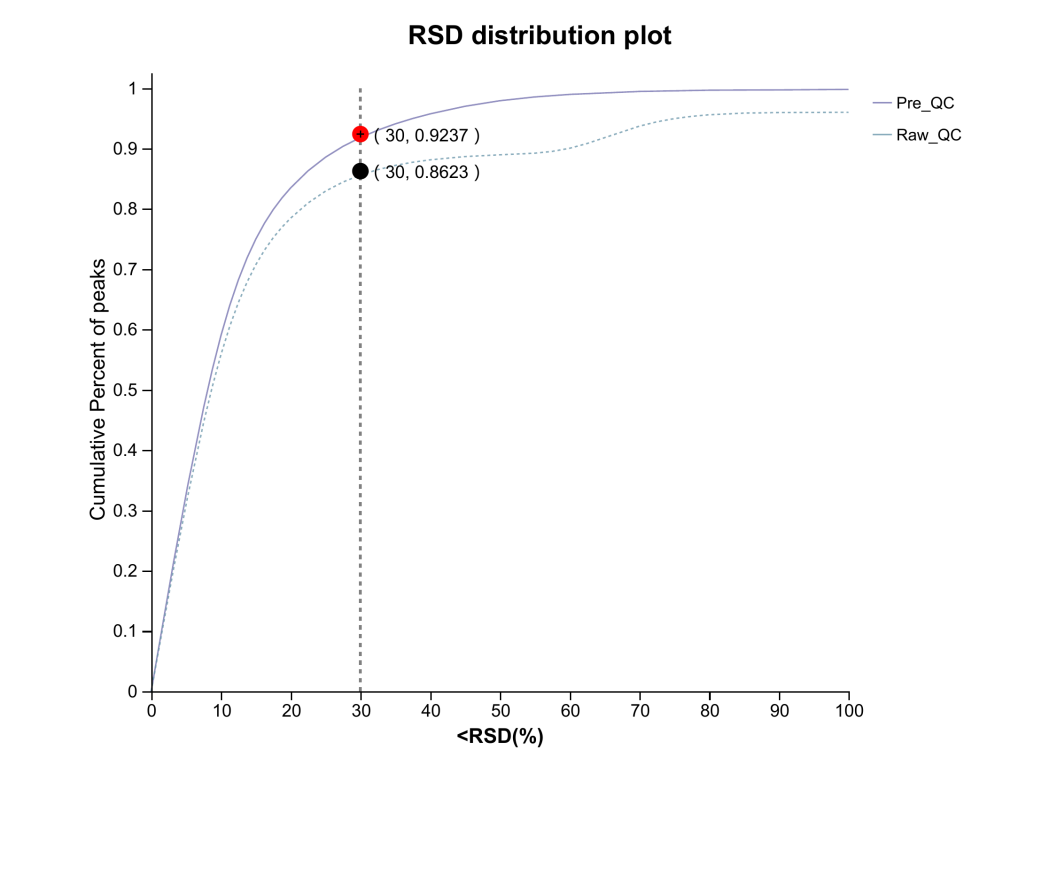


**Figure S2** QC-RSD Distribution Chart
